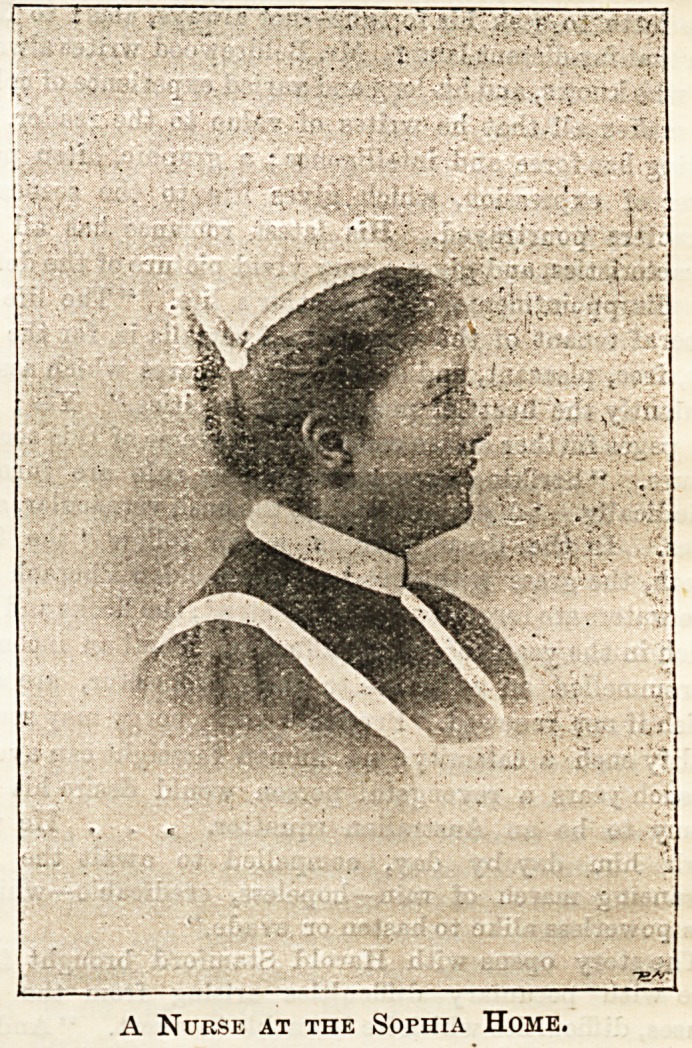# The Hospital. Nursing Mirror

**Published:** 1898-04-02

**Authors:** 


"The Hospital" Nursing Mirror.

Being the Nursing Section of "The Hospital."

[Contributions for this Section of "The Hospital" should be addressed to the Editor, The Hospital, 28 & 29, Southampton Street, Strand, London, W.C., and should have the word "Nursing" plainly written in left-hand top corner of the envelope.]

### News from the Nursing World.

### FACTORY GIRLS' COUNTRY HOLIDAY FUND.

With characteristic graciousness H.R.H. Princess Christian has consented to become president of the Factory Girls' Country Holiday Fund, in room of the deeply-lamented Duchess of Teck. This fund is responsible for sending 1,250 girls into the country or to the sea for something less than £1 a piece, of which they paid £246 themselves. Rapidly as the work is extending, it is only the lack of money that prevents the increase progressing by leaps and bounds. The action of the railway companies in not issuing half-price return tickets is much felt, especially as they have usually been generous to movements of this nature. They are, of course, well within their rights, but as theirs is also the right to be liberal, their popularity would in no degree suffer should they exercise it. Possibly their purse would not be lighter either. The claim such societies have is one that appeals to all. London is no fitting residence year in and year out for human beings. What is bad for one is bad for all, and we who watch with interest preventive as well as remedial medicine and sanitation, wish the work God-speed.

### THE FREE HOME FOR THE DYING.

Apart from the national and truly benignant work of the Free Home for the Dying, this charity has another claim upon the alms of the public: it has never been in debt. The only passport needed to open its hospitable doors is a medical certificate that the applicant is dying, and from his admission until he enters the dark portals of death all that science and sympathy can suggest is done to make his "passing" easy. It is a fact of considerable significance that visitors as a rule become subscribers. At present there are 16 beds, and efforts are made, and hopes entertained that the building fund will increase in order to provide more accommodation. How often human judgment may err is curiously illustrated by the fact that three of the moribund patients were discharged during the year having been restored to such a measure of health as to render them inelegible as inmates of the home; the address of which is 82, the Chase, Clapham, S.W.

### ST. PATRICK'S NURSES' HOME, DUBLIN.

The twenty-first annual report of St. Patrick's Nurses' Home, Dublin, lies before us. This is an entirely charitable institution, administering only to the sick poor, and every room and cubicle of the Nurses' Home, accommodating fourteen, is full. There is the lady superintendent, senior nurse, six staff nurses, and six probationers, for of late years these are trained for Queen Victoria's Jubilee Institute for Nurses in district work after their hospital course is accomplished. A Dutch lady, Miss Kruysse, was trained by the home in 1894 to take the post of district nurse at Rotterdam, and, as she has been appointed matron of the largest hospitals in Holland, another Dutch lady, Miss Van der Hoeven, is now under instruction at the home, preparing to take her place. The nurses made 36,061 visits during the year. With regard to funds, the accounts of the year began with a debt of £115 and a "friend's" cheque to clear it. They end with an overdraft on bankers for £1 only, out of a total of £1,701.

### THE CHELSEA INFIRMARY.

The Chelsea Guardians have just shown, in a substantial manner, their appreciation of Miss de Pledge's services as matron of the infirmary, by awarding her a gratuity of £25. Mr. John H. Brass, a leading member of the Board, in supporting the recommendation, concluded a highly complimentary speech in the following terms, which we reprint from the local press: "No one would question the capability and thoroughness of the Matron of the Chelsea Infirmary. (Hear, hear.) She stood second to no other matron in London. For the excellent staff now in the service of the Guardians they were wholly indebted to Miss de Pledge. They had spent £15 in unsuccessful advertising, and failed to get the staff they required. Then Miss de Pledge obtained the requisite nurses, and made the Chelsea Infirmary a model establishment of its kind in London. Therefore, as far as the gratuity itself was concerned, no one could say the Guardians were over-remunerating Miss de Pledge."

### TAUNTON DISTRICT NURSING ASSOCIATION.

For six months the banking account of the Taunton District Nursing Association has been overdrawn, and the treasurer has feared an adverse financial statement at the end of the year. However, shortly before it closed, some handsome donations placed matters in a happier state, and left a balance to the good of £15, the total income being £372. It was stated at the annual meeting that the Diamond Jubilee had been inimical to subscriptions, and that more contributors must be obtained before affairs were in a thoroughly satisfactory condition. There was some little heart-burning over granting £20 to the district of Rowbarton, as hitherto. The inhabitants of that place meet this grant by finding £50, so that the nurse receives a salary of £70. The difficulty arose from the fact that a Nonconformist minister did not like applying to the Vicar of St. Andrew's for a nurse. "The district fund," he urged, "was undenominational, but the grant to the Rowbarton nurse was not." He proposed as a remedy that the central association should nurse the borough. This, however, involved so many considerations, of which not the least is that of superintendence, that the meeting put off decisive action until next year, a rider only being added to the grant stating that it was made on condition that it should not be asked for again. Difficult as the matter is, it is pleasant to note that it is being dealt with entirely as a matter of principle, and not as one of personal feeling.

### RECOMMENDS.

The question of "Recommendations" to the services of the nurse was much discussed at the annual meeting of the Dukinfield Sick Nursing Association. The plan pursued by this society is to give, or rather sell, every subscriber of 2s. 6d. a "recommend," as it is familiarly called, and this course seems firmly rooted in the affections of the committee. The chairman (the Rev. W. Titterington) disapproved, and entered his protest against it in moving the adoption of the report. We share the chairman's views. Charity ought to be free. The sale and purchase of "recommends" introduces barter into bounty, and not always on strict business principles; for instance, by a funny anomaly the association on occasion can manage to sell the same "recommend" twice over, as when large subscribers do not take up all their privileges in this respect their "recommends" may be obtained from the hon. secretary for 2s. 6d. each. One gentleman suggested that the number issued should bear some relation to the capabilities of the nurse, as there must be a limit to her powers, and not to the number of subscriptions desired. We should also like to know how the committee would meet their engagements in the event of all the "recommends" being taken up and used, and the illogical position is this: any one can buy "recommends" to any extent of his or her supply of half-crowns, and is therefore in a position to demand the services of a nurse for the full amount. £80, for instance (the cost of a nurse), would buy 640. Such a number of cases for such a sum would mean bankruptcy to any association.

### A NEW DEVELOPMENT.

THE Whitby Nursing Association, established some eighteen months since, has proved so successful that it has been followed by the opening of a cottage hospital in that town. Mr. Beckett, M.P., opened the building, and spoke of the value of having such a hospital, offering facilities for the performance of operations and obtaining special treatment such as could not be had at home.

### FIVE YEARS IN LABRADOR.

THE trained nurse is becoming ubiquitous. Even the natives of snowy Labrador have for the past five years regarded Sister Cecilia Williams, of the "London," as their own special property. "You are one of us; you are our own sister; you know us; we cannot do without you now," is the touching appeal to their "own sister," and deep must be their disappointment when they learn that she is not to return this season. Much as she regrets having to relinquish this work, five years' battling with no ordinary hardship has not passed without leaving its impress on her health, and is not to be faced again with impunity, and for such work full vigour is needed.

### PROBATIONERS FOR NURSING MENTAL DISEASE.

SOME of the asylums of the Metropolitan Asylums Board afford an excellent field for young women in which to acquire the art of attending the mentally afflicted. At the School for Imbecile Children at Darenth, Dartford, for instance, healthy, trustworthy women of 20 are accepted as probationers; the medical officer gives them lectures on anatomy and physiology, and the matron teaches them general nursing. The wages are good, beginning at £17 a year and increasing to £19. The asylum is handsomely built, and stands high; the nurses have a beautiful sitting-room, whilst, thanks to the early training of the children, the work is not so repulsive as it is often imagined to be. The children's day rooms are bright with flowers and brightly-coloured tablecloths; their dormitories are also spotless and attractive, whilst they themselves are often affectionate, attaching themselves to their teachers and attendants; and, although there is very little hope of cure, most of them are taught many things. The matron is a trained nurse of wide experience, and encourages her nurses to make their own room comfortable and homelike, and to improve themselves. This somewhat long account is given because queries upon this subject have been somewhat numerous of late, and also because two years' valuable and remunerative training in the care of imbeciles can be taken before candidates are of an age to enter a general hospital, therefore the information will probably be useful. It may possibly have struck some more or less enterprising damsel that training in the care of this class of disease, combined with that of general nursing in a children's hospital, might open the door to some lucrative appointments in private work, for imbecility is not exclusively to be found amongst the poor.

### SEATS.

THE much-vexed question, Shall the shop assistants be provided with seats? is now becoming one of nursing import. Continuous standing is most injurious, yet, as a writer in *Guy's Hospital Gazette* points out, whilst the medical officers on duty are permitted to sit down whenever the opportunity occur, sisters and nurses must remain on their feet. Of course, it would never do for a nurse on duty to subside on to any support that presented itself. It is impossible to imagine the confusion in a ward wherein the sister-in-charge seated herself gracefully on the nearest bed or locker, whilst the probationers perched themselves gaily on tables and window seats. The whole thing is subservient to all ideas of propriety, and well-trained nurses would scorn it. There is also another point which is overlooked. Whilst in ninety-nine cases out of a hundred the medical officer has nothing to ask and no instructions to give, yet in the hundredth a phrase, an expression, may give the nurse the clue to the treatment desired, which is not always conveyed in the most minute directions. The nurse, it is evident, must remain "at attention." How can she do so, and sit down as much as possible, is a problem still to be solved.

### SHORT ITEMS.

THE term of training probationers at the Royal South Hants Infirmary, Southampton, has been increased from two to three years. — The Bishop Auckland Nursing Association has decided to ask for subscriptions in order to provide a home for the nurses, a project held over from last year on account of the many Jubilee claims.—Nine young nurses are being trained by the Cornwall County Association for district work, and until they are ready for work a certain number of competent nurses have been engaged. The lady superintendent is busy giving lectures and nursing.—Miss Emma Halford is gazetted with the Royal Red Cross decoration.

### Antiseptics for Nurses.

By a Medical Woman.

### VI.—COMPOSITION AND USES OF SOME OF THE ANTISEPTICS IN COMMON USE.

Iodine—Iodoform—Eucalyptus—Thymol—Boracic Acid—Salicylic Acid—Sanitas.

If iodine had not the great disadvantage of staining both the skin and linen brown it might be far more extensively used, as it is a very powerful antiseptic.

From iodine, however, is obtained that well-known antiseptic, iodoform. It occurs in two forms, a bright yellow crystalline substance, which dissolves readily in chloroform and in alcohol, but is quite insoluble in water; and a fine yellow powder. The crystalline form is said to be best for large wounds or raw surfaces, as it is less readily absorbed than the powder. Iodoform is very volatile, and has a very penetrating odour, which some patients find so extremely disagreeable that it cannot be used for them. At first iodoform was very popular with surgeons, but now it has been found to possess serious drawbacks, and Erichsen says of it, "According to the ordinary modes of testing antiseptics, iodoform is practically useless, but it is one of our most valuable means of preventing putrefaction and infection." This discrepancy is said to be explained by the fact that iodoform breaks up in the presence of pus, so that pure iodine is liberated. Free iodine is well known to be one of the most powerful of all antiseptics, and thus the power of iodoform in checking decomposition and suppuration is fully explained. Fine iodoform powder is said to prevent blistering of the skin round a wound under the dressing if powdered over the skin.

The preparations of iodoform are gauze, which should contain 20 per cent. of iodoform, prepared by rubbing iodoform into sterilised gauze and fixing the iodoform with glycerine; iodoform wool; Sir Joseph Lister's emulsion, which is made by adding 10 parts of glycerine to 1 part iodoform, and some surgeons prefer the further addition of 1 part of ether and 2 parts alcohol; and iodoform lint. Iodoform is also made into bougies with cocoa butter.

Eucalyptus was introduced about 1880, and was in great favour at first, and was said to be three times as strong as carbolic acid, and to prevent putrefaction better than any other antiseptic. Although this high promise has not been fulfilled, eucalyptus is an excellent antiseptic, and it has the great advantage of having a pleasant, fragrant odour, and it is non-poisonous, so that its absorption does not cause any toxic effects. It dissolves easily in oil or alcohol, but not in water. The preparations of eucalyptus are gauze, which should contain 50 per cent. of the antiseptic, which, however, is extremely volatile; and ointment, made with 1 part eucalyptus to 2 parts each of paraffin and vaseline, which gives excellent results.

Thymol is obtained from oil of thyme by cooling, when thymol separates out in crystals. It has the agreeable odour of thyme, and has no irritating or poisonous properties, and when first introduced it was in great favour for a while, but it did not answer the expectation entertained, as it was subsequently found that it did not arrest putrefaction. It is very soluble in alcohol, but not in water. Thymol gauze is prepared, but thymol in any form is very rarely used now as an antiseptic.

Boracic, or boric acid, is a white crystalline powder, which is obtained from the volcanic borax from Tuscany by refining. It has a somewhat alkaline taste, has no odour, and is freely soluble in water. It is a weak antiseptic as compared with carbolic or corrosive sublimate, but ranks with eucalyptus, salicylic acid, creolin, &c., as a non-poisonous antiseptic. Boracic acid was first introduced by Sir Joseph Lister, and is still very largely used as a lotion. A cold saturated solution contains 1 part boracic acid to 26 of water, but a much stronger solution, viz., 1 part to 3 of water, can be obtained by using hot water. It is very useful when an antiseptic is required for a large raw surface, or for washing out cavities, when there might be danger of absorption if a stronger and therefore poisonous antiseptic were used.

Its preparations are boracic lint and wool, which are prepared by soaking in saturated solution and drying. The pink colour is not in any way connected with the boracic, but is due to a perfectly harmless vegetable dye (litmus), and the object in colouring is to distinguish boracic from other preparations. A boracic ointment is prepared containing 1 part boracic acid to 5 parts of the usual base, but this ointment is not much used. Boracic is now largely used as a preservative for foods, as boroglyceride, which is merely a mixture of boracic acid and glycerine, the boracic acid being added to the point of saturation.

A patent antiseptic called "Listerine" is sometimes met with; it is another form of boracic acid, to which is added oils of thyme, eucalyptus, menthol, &c.

If there should be any doubt whether a certain white powder is boracic acid, it can be easily settled by dissolving a little in some methylated spirit or alcohol in a saucer and setting a light to it. If it be boracic acid the flame will be of an unmistakeable green colour.

Salicylic acid is derived from carbolic acid, and is closely allied to it. There are two varieties of salicylic: 1, Natural, which is derived from oil of water-green; 2, artificial, which is more toxic in action than the natural. It has been combined with almost every metal, but the form most commonly used is sodium salicylate. Oil of wintergreen is a colourless fragrant fluid, very soluble in alcohol, but not so soluble in water, and possesses no toxic properties. Salicylic acid does not coagulate albumen, is non-corrosive and non-poisonous. It is soluble in water in the proportion of 1 part salicylic to 300 parts water, and in this strength is a less certain antiseptic than carbolic acid, and less irritating, but it affects the hands like carbolic, and corrodes steel instruments. It is also absorbed and occurs in the urine of the patient like carbolic acid. The strong alcoholic solutions of salicylic acid have caustic action, and hence are used for corns on the feet. The preparations are: Salicylic wool, containing from 2 to 10 per cent. of salicylic acid. The wool should be treated with glycerine to prevent the crystals of salicylic acid from flying about when the wool is used and irritating the throat. Salicylic jute is the bark of a special tree prepared in the same way as salicylic wool. The advantages of jute are that it is cheaper and more absorbent than wool. Salicylic gauze is prepared by soaking gauze in a solution of salicylic acid, glycerine, rectified spirits, and distilled water. Salicylic acid cream is a mixture of glycerine and salicylic in proportion to form a cream. One of the best known derivatives of salicylic acid is salol, which is a white crystalline powder with a faint aromatic odour, tasteless and insoluble in cold water. Salicylic acid is used as a preservative of foods.

Sanitas is one of the essential oils, and is obtained from oil of turpentine. The active principle is camphoric peroxide, and its action is that of an oxidiser. There are two forms of sanitas—sanitas fluid, with a pleasant aromatic odour, and of a light straw-colour, and sanitas oil, which is a brown, syrupy liquid, lighter than water. It is soluble in alcohol but insoluble in water, and has an aromatic odour like the fluid. The preparations include the "Sanitas air-purifier," made of sawdust soaked in camphor peroxide. Sanitas is used extensively in soaps, washes, &c.

### Post-Graduate Clinics for Nurses.

By a Trained Nurse.

### XLIII.—THE NURSING OF PNEUMONIA.

At this season of the year pneumonia is always more or less prevalent, and more especially when influenza is "in the air," so that a few hints on the nursing of patients suffering from it may prove timely. Pneumonia may be an independent disease, a complication arising in the course of many illnesses, or may accompany the puerperal state and a septicæmic condition. Its onset may be gradual, or may immediately follow sleeping in a damp bed or exposure to chill. It is a disease which calls for skilful nursing. Warmth and feeding, accompanied with perfect rest of mind and body, are the more important features in the nursing of pneumonia. A pneumonic patient should invariably wear a flannel nightgown, cotton or linen next the skin being absolutely inadmissible in the nursing of any type of lung disease.

A wadded foot cosy, or a loose flannel bag, should be used to prevent cold feet, either of these being preferable for constant use to a hot-water bottle. For the first two or three days however after the rigor, and to counteract general shivering, a hot bag in the bed is advisable and even necessary.

As to the temperature of the room doctors differ; all, however, agree that ventilation must be thorough, and that the temperature ordered must vary as little as possible in the twenty-four hours.

The ordered temperature of the room will depend much on the age and temperament of the patient, and on the amount of stimulation and food he is receiving. Steam kettles are sometimes employed in pneumonia; 67 deg. to 68 deg. is the highest average in England, while in the United States 70 deg. to 72 deg. is the favoured temperature of the air. But many English doctors prefer from 62 deg. to 65 deg.

Bedroom fireplaces often prove most unsatisfactory, and are sometimes so constructed that most of the heat goes up the chimney, so that a nurse often finds it very difficult to maintain even so low a temperature as that of 63 deg. But she must not relax her efforts to overcome the disadvantages of such aggravating fireplaces.

So far as pneumonia jackets are concerned, I find that square sanitary towels sewn together, the outer part covered with oil-silk or thin jaconette, serve admirably. Put on after well warming and "fluffing" before a fire these are most comforting, being light, and therefore causing no oppression or hampering of the chest movements. I must enter my individual protest against splitting up the nightgown at the back, unless the back is to be poulticed, when of course it is protected.

Linseed poultices are rarely used now in pneumonia, unless this be complicated with bronchitis. Mustard leaves, leeches, and blisters have replaced the old poultices. Risk of chill undoubtedly attends poulticing, but a careful nurse can guard against this danger. One plan which is sometimes used to obviate risk of surface chill whilst the poultice is being changed is to keep on a flannel jacket, over which the jacket poultice is applied. Personally I have found this remedy worse than the disease, since the flannel jacket absorbs all the damp of successive poultices, and "poultice rash" may consequently be produced. Ice-bags are frequently used on the chest, this form of treatment being preferred by some doctors to hot applications. In applying ice the nurse must be careful not to allow the bag to remain on the chest after the ice has melted.

As most nurses know, the onset of pneumonia is frequently accompanied by a rigor and bad headache, or in children perhaps with vomiting and convulsions. The first stage may last from two to five days, after which symptoms of lung consolidation set in, this second stage being characterised by high fever and increased consolidation of the lung substance. In a favourable case at the stage of crisis the temperature falls rapidly, and convalescence is soon established. When the crisis takes place profuse sweating, causing increased tendency to chills, and a diarrhœal condition may set in. In the latter case the nurse should apply a flannel abdominal binder and carefully guard against cold feet.

In the fever stage the skin is hot and burning, the patient prostrate, temperature about 104 degrees, dyspnœa, and blood-stained sputa present. If the sputa be prune-juice coloured it is a bad symptom. The pain in the base of the lung—or in both lungs—may call for the application of a blister. A four-inch blister with a linseed poultice put on immediately after may be ordered. During the fever stage the nurse should pass a little warm sweet oil on a piece of cotton up the nostrils at intervals, this simple measure soothing and relieving the sore and inflamed nasal passages.

Excitement and muscular exertion may prove fatal from their effect on the heart, weakened, as this is, by the additional work thrown upon it by the partial solidification of the lung and the depressant effect of high fever. No sitting up should be allowed, and talking should be strictly limited, especially at the crisis of the disease, when the patient is usually very prostrate. Any appearance of lividity of the finger-nails or round the lips and over the ear-tips should be noted, and, if severe, the doctor must be sent for.

The strain on the heart caused by interference with respiration, owing to the blocking of the air passages, is frequently very severe. Some patients fall into deep, heavy sleep, during which the mucus, being uncoughed up, tends to block the air passages to a dangerous extent.

The feeding of a pneumonia case is an important question, more especially when the heart shows signs of strain. Nourishment should be given every two hours. Some nurses have the tendency to give pneumonia—and other sick people—too much fluid. In a desire to nourish the patient it is sometimes forgotten that quality, rather than quantity, is an important factor.

Serious gastric disturbance sometimes accompanies pneumonia, and peptonised foods may be necessary. Cream, whipped or raw, should form a large factor in the feeding of pneumonia, and if pure milk be given it should be previously boiled and flavoured with tea, coffee, &c. Whipped eggs, chicken broths, peptonised cocoa, and beef-tea may "ring the diet changes."

The port wine and stimulants ordered will vary according to the age and condition of the case, and should always be given with or immediately following food.

So far as the ward-nursing of pneumonia is concerned, it should not be forgotten that pneumonia has an undoubted quality of infectiveness. In the hospitals of the United States it is an unwritten law—strictly attended to—that cases of pneumonia shall not be placed in beds close to those of infants or delicate child patients. In fact, they are isolated to some degree. In private cases little children should not be allowed to sit on the beds of pneumonic patients, nor should such patients kiss their young children. A case of a pneumonic mother insisting on having her ten-month baby constantly with her during her illness came under my notice a month or so ago. Without otherwise pointing the moral of cause and effect, it is a fact that the baby contracted the disease and died.

### Nursing Developments in London Institutions.

PROGRESS DURING 1897-1898.

THE LONDON TEMPERANCE HOSPITAL.

The very latest addition to the building is a nurses' home, a house adjoining the hospital having been purchased, altered for the purpose, and fitted up in the most generous possible way, as a gift to the hospital, by Mr. and Mrs. Geo. Cadbury in memory of the former's mother (Mrs. Taylor), one of its earliest friends. The new nurses' home provides a charming dining and sitting room for all the staff and six additional bed-rooms. The dining-room is an addition built between, and connecting, the hospital and the home. It is a cheerful room, lighted by a large top light as well as by two windows, the former being shaded, like a ship's cabin, by pale green blinds drawn horizontally across. The furniture is stained a pretty dark green, the long tables having serge covers of a dark red. The sitting-room is divided by curtains, and is filled with cosy chairs and sofas and pretty things, even to a piano. There is a kitchen, with a stove, in the basement, where nurses can make their tea when they wish, and a bath-room. Upstairs the bed-rooms are truly dainty little apartments, furnished with every regard to the nurses' comfort, even to the extent of a pretty framed print in each room. The home is lighted with electric light. One of the wonderful "Gorham" beds completes the inventory. The object of this ward is to provide a room for major operations from which the patient need not be moved.

ST. THOMAS'S HOSPITAL.

At St. Thomas's Hospital Miss Gordon and her nurses are to be congratulated upon the addition of new quarters for the night staff. The house formerly apportioned to the chaplain has been cleverly adapted and arranged as a home for twenty-one nurses, giving to each a separate bed-room. There is a snug sitting-room, furnished most comfortably, and here the nurses have their lunch before going to bed, two maids being told off to the care of the house, with a bed-room, sitting-room, and kitchen for their special use. The bed-rooms are fitted up admirably. Each contains a hanging cupboard with long glass, a washstand with cupboard and brass swinging towel-rail, dressing table with three long drawers and two small ones, small table, book-case, and chair, and white-painted bedstead. The furniture is of polished pine, and the crockery of an especially pretty pattern and colouring. The furnishing has been done by Messrs. Shoolbred, the easy chairs in the sitting-room coming from the Bentwood Company. The sanitary arrangements have been all renewed (and in the hospital dormitories as well as in the new home), the slop-sinks and and w.c.'s being furnished by Dent and Helyar and by Doulton. The house is heated by hot coils, and lighted throughout with electric light. There is a good provision for boxes and trunks, and an "airing-room" in the basement.

QUEEN CHARLOTTE'S HOSPITAL.

Considerable alterations and additions are in course of completion at Queen Charlotte's Hospital. The building has been heightened by a new floor devoted to the accommodation of sisters, while that immediately beneath—formerly used for this purpose—is being made into new wards. These will not be ready for use yet awhile, but the sisters' rooms above have been completed and are in use. They are large bed-sitting-rooms, extremely airy and comfortable, with parquet and carpeted floors, and very nicely furnished. Two or three charge nurses have also rooms on this floor, and there are baths and lavatories. The addition of a linen-room and also of a cloak-room, where nurses coming across from the home can leave bonnets and cloaks, make greatly for the comfort and convenience of the nursing staff.

### The Association of Asylum Workers.

SIR JAMES CRICHTON BROWNE took the chair at the annual meeting of the Association of Asylum Workers, which was held at the rooms of the Medical Society, 11, Chandos Street, at 4 p.m., on Monday, March 28th. He opened the proceedings by moving the adoption of the report in an able and scholarly address lasting three-quarters of an hour, during which it was frequently evident that he had the full sympathy of his audience. This address, which will be presented verbatim in the next issue of the *Asylum News*, is very full of interest to nurses of all grades. He maintained that the mental nurse was second to none, nay, superior to most. She must first be a nurse, having at least one year's training in a general hospital or in the infirmary wards of an asylum, and then she must possess tact, self-control, patience, and strength in a marvellous degree to be successful in this arduous undertaking. The question of pensions should, he said, be definitely settled, and not left to the tender mercy of county councils. He regarded frequent and ample holiday as necessary, and thought that asylum workers ought to get as far away from professional companions and topics as possible. He did not think a University degree the best method of finding just the women most suitable for the work, an opinion that Mrs. Creighton, wife of the Bishop of London, who seconded the motion, contested. The special resolution submitted, "That the meeting approves the provision for asylum workers in sickness either by a home or other means" was proposed by Dr. Mocatta, whose advice to workers with a month's holiday in the year was to take it in two parts of a fortnight each. The Rev. Henry Hawkins seconded the resolution, and it was carried. Sir James Crichton Browne has consented to be president for the ensuing year in place of the late Sir Benjamin Ward Richardson. The name of the Bishop of London is added to those of vice-presidents. Dr. G. E. Shuttleworth succeeds Dr. Walmsley as honorary secretary. A vote of thanks to the chairman closed the proceedings.

### The Royal British Nurses' Association.

WE are authorised to state that Dr. Bezly Thorne has withdrawn his intention to resign from membership of the Royal British Nurses' Association. The Hon. Treasurer, in making this satisfactory announcement, expressed his unqualified approval of the manner in which Dr. Bezly Thorne and Miss Grace Gordon discharged their administrative and official duties as Medical and Nurse Hon. Secretary respectively, and alluded to the vote of confidence in which this approval was embodied on the minutes in 1894. The Medical Hon. Secretary spoke in warm terms of the kindness rendered to him by Dr. Bezley Thorne on the occasion of his taking over the duties of Medical Hon. Secretary, and expressed his pleasure in the continuance of Dr. Thorne's co-operation in the future development of the Association, since few understood its aims better or had worked harder in its service.

### WALSALL AND DISTRICT HOSPITAL.

THE first annual ball was inaugurated by the matron and friends for the benefit of the above hospital. It was held in February in the Assembly Rooms, and proved a great success, the institution benefiting thereby to the extent of £114, which the executive committee have decided to set apart as the nucleus of a building fund for providing better accommodation for the nursing staff.

### Novelties for Nurses.

### NOVELTIES FOR NURSES AT THE PRINCIPAL DRESS HOUSES.

Notwithstanding the cold and inclement weather which has characterised the early days of spring, the shop windows are beginning to look very smart, and indications are not wanting of preparations for brighter days at Easter. When the warm weather does come it will probably come suddenly, and unenviable will be the lot of those who have not anticipated the change by the addition to their wardrobe of lighter clothing. Nurses, like other people, require to study the seasons, and they will find at few establishments such a varied and high-class assortment of novelties as at that busy emporium in the Edgware Road owned by Messrs. Garrould. Here nurses are abundantly catered for, and all their requirements carefully studied. Particularly attractive is their selection of caps, of which there are a large number on show. The "Albeville" is an extremely elegant design, and will be found from its shape to be generally becoming. There are also some becoming uniform gowns, among which the "Princess" is sure to be a favourite. The boot and shoe department deserves special mention. Messrs. Garrould have given it their careful consideration, with the result that their shoes will bear comparison in point of comfort and elegance of shape with the best houses in the trade, and at very moderate prices. The "Sickroom" slipper is quite a marvel of ingenuity, and can now be procured with a layer of felt inside the twine sole which renders them exceptionally durable. In dress materials there is a large assortment, and many of the designs are really beautiful. A linen-finished cloth, called "Halifax," is very fascinating, and would serve many other purposes equally well with the one for which it was primarily intended. Temperature charts for nurses are a specialité of this enterprising firm, and can be had at 4½d. the dozen. "Benton's" diet charts are the same price; cases can be had singly at 6d. each, or double-folding at 1s. each. At Messrs. Debenham and Freebody's in Wigmore Street, there is a department set aside specially for nurses, where a complete outfit may be obtained at a reasonable cost and without any trouble. We always admire the cut and style of the cloaks turned out by this firm; they are in such good taste, and look well to the end. There are one or two new shapes which are likely to become popular. The "Norah" is one, and the "Dora" is another, though this latter can hardly be described as new, though it will always continue to be a favourite. The "Victoria" will be found most useful to nurses who cycle, for which purpose it has been specially designed. It consists of a close fitting under-garment buttoning down the front, with a certain amount of fullness at the back and a detachable cape. A neat little bonnet goes with it, slightly peaked in front, and trimmed in front with a full bow of ribbon. There are several pretty bonnet shapes, some with veils, according to the taste of the wearer. Very charming are the dress materials, especially the Irish linens, which are to be had in all colours at the modest price of 1s. 2d. per yard. Linen aprons are offered at all prices, according to quality and workmanship, from 1s. 6d. upwards, and excellent they are both in shape and texture. Instruments also and travelling bags are kept in great variety, also furniture suitable for nurses' bed-rooms in most useful designs. Of the furniture we are specially qualified to speak, and can pronounce it most emphatically to be in every way admirable. At J. R. Roberts' Stores (Limited), 96, Stratford Broadway, is to be seen a delightful surgical wagon or table. The framework is satin walnut, and the shelves, which are loose and detachable, are of plate glass. This will be found a boon in a surgical ward, as much for the ease with which it can be moved about from place to place as for its cleanliness and aseptic properties. This firm also makes a study of nurses' requirements, and always keeps in stock a well-assorted selection of cloaks, bonnets, capes, aprons, and materials suitable for washing dresses. In Oxford Street we find D. H. Evans and Company have opened a department for nurses. They are showing at present an excellent-shaped linen apron at 2s. 6d., and also some pretty designs in cuffs and collars. We greatly admired a well-fitting cloak, called the "Clipper," which is made in either navy or black Heptonet cloth, at a cost of 18s. 9d. There were also some comfortable-looking ward shoes, and a delightful "silent" slipper with thick felt soles. Harrod's Stores are also well to the fore with novelties, and have some lovely designs in washing material which it would be difficult to surpass. They have recently brought out some dainty hemstitched linen cuffs and collars suitable for a matron's wear, which we have every confidence in recommending. There are also other patterns equally choice, which have only to be seen to become popular. The "Mildred" is a shower-proof cloak of elegant shape, which can be had in either navy, black, blue, or grey, and varies in price from 21s. to 29s. 6d., according to the quality and thickness of the cloth. A neat little trunk, suitable for travelling either at home or abroad, is worth inspection, and would prove a useful receptacle for the many purchases which a visit to this fascinating establishment perforce entails.

### NOVELTIES AT MESSRS. EGERTON-BURNETT'S.

A box of patterns from the well-known Wellington firm is always a liberal education, as in a measure it anticipates the fashion. So many and varied are the samples that it would seem almost impossible to improve on them, and yet each season brings with it some fresh novelty, some further improvement either in design or texture of already favourite fabrics. Though the general public are abundantly catered for by Messrs. Egerton Burnett, they have not overlooked the needs and necessities of nurses, and we have much pleasure in drawing the attention of our readers to the really charming assortment of serges, beiges, and waterproof material in improved designs suitable for spring or summer wear. The "Royal Serge" is always a fascinating production. It has the merit of being inexpensive and is everlasting wear. In either navy blue or black it cannot fail to be popular; the colour is good, and it is warm as well as light, a great consideration to those who have to be out in all weathers. For bicycle riders the serge will have great attractions, though the cloakings and covert coatings will run it pretty close. There is white wincey that promises to become popular; it is very fine and light, and washes beautifully. Nurses will more especially welcome patterns of new washing materials, galateas, shirtings, and zephyrs vie with each other in beauty of texture and colour, so that it is hard to say which material takes the palm. There are some delightfully fresh looking blues, plain and striped, that will find admirers, and a delicious soft shade of grey suggestive of the Quaker costumes, so quickly, alas, dying out. A good serviceable linen, 40 inches wide, is to be had at the moderate cost of 1s. 7½d. per yard, and there is also a clear cambric that would prove ideal material for caps. So many and varied are the patterns that it is impossible to do justice to them in the limits of a review, and those of our readers whose curiosity is aroused will in no way be disappointed if they write forthwith to Messrs. Egerton Burnett, Wellington, Somerset, for a box of their latest spring patterns, which will be sent post free.

### Nursing in Paris Hospitals.

### A.—LAY NURSES.

### VII.—The Question of Lodgment.

The somewhat bull-in-the-china-shop fashion in which the Municipal Council has laicised the nursing system in the Paris hospitals has raised many unforeseen and peculiar difficulties. In Catholic countries the natural element for recruiting guardians of the afflicted is in the monastic orders, where the same spirit which gives up the world for a religious life also inspires heroic sacrifices in aid of our fellows. Far be it from me to say that the element is anywise less in Protestant lands. As a fact, especially in womankind, this spirit, of devotion is quite as diffused, and is pre-eminently shown in the direct enlistment in hospital works, without the preliminary formula of the religious vow. Moreover, in England there is in the nursing service a far larger proportion of women of refinement, culture, and social position, who have sacrificed everything from a desire to do noble, if lowly, work for others, than in the ranks of the French nurses, either lay or religious.

In breaking rudely with immemorial tradition ignoring the powerful social influence which had always furnished the hospitals with nurses, and undertaking to recruit the female portion in the ordinary run of women, the Municipal Council soon found the question of celibacy all important. The religious Sisters of the Catholics are the equivalent of the hospital devotees of the Protestants, knowingly foregoing the enjoyments of domestic life among other comforts foresworn. For them husband and children are out of the question. Not so with the lay nurse, who merely raps at the hospital door for employment as she would apply to a household as housemaid, or to a tailor's shop as a seamstress. Almost invariably the new lay nurse enters the hospital because she has no other resources for the time being. Such a recruit has ordinarily no intention of living a life of "single blessedness," if she receives an offer of marriage at all to her taste. Such marriages place the administration on the horns of a dilemma. Either the nurses leave the hospital at marriage and injure the efficiency of the staff by frequent changes, or provision must be made for family life of some kind.

If nurses were like ordinary non-domestic wage servants, of course the question of celibacy or married state would not trouble the administration, so long as each nurse appeared at the hospital at the proper hour and worked faithfully until the hour for ceasing work. As a fact, however, nurses are expected to be at hand at all hours, to be called upon for any emergency. Still there is a great diversity of opinion on this subject among the Paris hospital chiefs. The directors of the Hôtel Dieu, Saint Louis, La Charité, Laennec, Tenon, Ricord, Hérold, all agree in objecting to having nurses lodged in the hospitals at all; on the other hand, the chiefs of La Pitié, Cochin, Beaujon, Necker Lariboisière, Enfants Malades, Broca, La Maternité, are all in favour of having nurses live on the premises. Some of the reasons for these opinions are peculiar; several who favoured inside lodging agreed with the idea of M. Baron, the director of Cochin, who said that in case of any emergency, such as a great public catastrophe in the night, it would be a very fatal arrangement to have to send to distant lodgings for the extra nurses needed, and nurses coming from such lodgings, summoned without any expectation, would be in a poor condition to at once begin their work.

Curiously enough in all three hospitals near the Bastille I found the directors had all mixed opinions. M. Mulheim, at Saint Antoine, and M. Vallin, at Andral, would have the single nurses inside and the married ones out; while M. Valet, at the Trousseau Hospital, would have the upper grades out and the lower grades inside.

The Municipal Government are evidently partisans of the system of lodgments for the nurses on the premises, for they have begun an elaborate series of improvements in Paris hospitals for furnishing attractive, if not too ample, homes to the upper nursing staff, the grades which replace the nursing Sisters. There is now just being finished, and indeed partly occupied, in the Impasse de l'Enfant Jésus, a handsome stone block of six floors, with 53 separate apartments for the higher grades of the Hospital Necker and the Enfants Malades, the two establishments occupying the adjoining vast quadrangle at the juncture of the Rue de Sèvres and the Boulevard Montparnasse.

These apartments are of two sorts, having three rooms and two rooms each; the former are for the head nurses and under heads, the latter for the assistants. One of the rooms in each class is very conveniently arranged as a kitchen, with range, &c. The apartments are altogether very desirable, and are already much desired, there being a great eagerness among the staff to get allotted to one of the new quarters.

Probably the Enfants Malades was selected as the scene of this first attempt on account of the notorious character of the nurses' old lodgment, the building replaced and about to be destroyed having been vulgarly called the "Flea-box." Most of the nurses' lodgments are but little better, and at La Pitié the case is even worse. It is, however, not the upper grades, but the lower grades, male and female, whose lodgment is the chief scandal. They are crowded into miserable dormitories under the roofs, some of which have neither warmth, water, nor water-closets. There is no privacy during the twenty-four hours, and in some even no cupboards. The demoralising effect of such surroundings can hardly be exaggerated. One foul spirit is almost bound to infect the rest. Women living in so vulgarising an atmosphere can scarcely be ideal ministers to the sick and afflicted.

Besides the Necker-Enfants Malades attempt at reform, the administration has started a few others. At Cochin, and in one of the new portions of Saint Antoine, half partition chambers have been given to the lower grade female nurses. The chief reason given for housing the lower grades in the hospitals is to keep them from immorality without; but it seems to me the whole housing system has been an inducement to immorality within. Whether the new system will work well remains to be seen. There is no restriction in the number of children in the lodging, except that they must be minors; but in two rooms, with a full grown boy and girl, as I have found instances, proper living seems impossible. Again, the mixing up of the nurse's own hospital allowance with the purchases for husband and children is a constant inducement to attempt at small peculations, of which many of the directors are well aware.

The whole lodgment question is far from settled. All the hospital directors express great repugnance at any interference with the inside of the nurses' lodgment. If the chief tenants appear at the proper hours and perform their duties satisfactorily, they may do pretty much as they like in their little home, which is as much their castle as the proverbial residence of an Englishman. Still, there is considerable difference. The non-limit to the number of children and full-grown boys and girls, in conditions conducive to immorality, is a much more serious question than the small pilferings for family use, and almost as important as that of the abominable dormitories of the lower grades. The misuse of the stores is almost always condoned on the plea that the children really need the food or the raiment, and no one could have the heart to deny them. There seems to be a tacit understanding that the husband of a hospital nurse is a negligeable quantity as a bread-winner.

Edmund R. Spearman.

### Diet for Invalids.

### FOOD FOR THE GOUTY.

The diet ordered for sufferers from this trying malady usually consists chiefly of beef, mutton, chicken, game, fish, eggs, green vegetables, stale bread, and a small amount of butter. All foods containing much sugar or starch must be steadily avoided by the majority of patients. The few may find they can take fruit, if not very sweet, and certain vegetables and puddings without ill-effects. Tea and coffee should be taken in moderation, and cocoa made from the nibs is best. A little lime juice put into any mineral water and sweetened with saccharine makes a safe and delicious drink. Cream in place of the lime juice is very nice. Messrs. Callard and Co., 65, Regent Street, supply bread, biscuits, and the special farinaceous food suitable for gouty patients.

The following menus for a week will be a guide to those who have to cater and care for this class of patient.

### Menus.

Sunday.—Breakfast: Cocoa made from nibs; lamb's sweetbreads fried; dry toast. Dinner: S'oubise soup; roast pigeon, bread sauce, potato chips; celery, biscuits, and cheese. Supper: Tomato omelet; gluten bread and butter, watercress.

Monday.—Breakfast: Coffee with cream; haddock on toast; whole-meal bread. Dinner: Consomme; fillets of beef, horseradish, spinach; cheese, custard pudding. Supper: Fish soufflé; ham sandwiches; dry toast.

Tuesday.—Breakfast: Tea; hot bacon and eggs; dry toast. Dinner: Roast chicken, stewed celery; lemon jelly sweetened with saccharine. Supper: Potted meat and endive; gluten bread and butter.

Wednesday.—Breakfast: Cocoa made from nibs; fillets of plaice; dry toast. Dinner: Celery soup; mutton cutlets, tomatoes; almond pudding. Supper: Chicken salad; brown bread and butter.

Thursday.—Breakfast: Coffee with cream; grilled ham and mushrooms; dry toast. Dinner: Spinach soup; roast lamb, mint sauce; potato croquettes; rhubarb fool and cream. Supper: Eggs with piquant sauce; gluten bread.

Friday.—Breakfast: Tea; fried smelts; dry toast. Dinner: Roast game, bread sauce; winter greens; cheese fritters. Supper: Minced mutton on toast; gluten biscuits.

Saturday.—Breakfast: Cocoa made from nibs; grilled game; dry toast. Dinner: Tomato soup; beef steak, potato straws; rhubarb jelly with cream. Supper; Shrimp soufflé; almond biscuits.

### Recipes.

Haddock on Toast.—Take a small or half a large smoked haddock and remove all the meat from the bones. Chop it very finely, and if you have half a pound, put it in a stewpan with an ounce of butter, a little chopped parsley, and two tablespoonfuls of thick cream, and a pinch of pepper. Make it quite hot on the fire, and stir with a wooden spoon. It will only take a few minutes to cook; in fact, when it is all hot through it is done. Turn it out on to dry toast, and garnish the edge with some nicely cut pieces of the same, and sprinkle the fish with coralline pepper. Serve all as hot as possible.

Fillets of Beef.—Take about one pound of fillet of beef and cut it into thick slices about half an inch; trim them and season each with pepper and salt, and a little chopped onion and parsley; butter a frying pan, lay the fillets in, and fry them quickly over a brisk fire; they will take about five minutes. When a nice brown colour take them up carefully on a slice, and dish them on a purée of spinach. Pour the following delicious sauce over them: Fry together one ounce of butter, the same of flour and tomato, till a deep brown, then stir on to them one pint of good stock (hot); stir till it boils, and simmer for fifteen minutes, add half a glass of sherry and a teaspoonful of lemon juice. Strain the sauce and pour over the fillets; this is best made ready, also the spinach purée, before commencing to cook the fillets.

Chicken Salad.—Take any remains of cold boiled or roast chicken, free the meat of skin and bone, and cut it into Julienne strips; well wash some celery and cut it in the same way; drain and dry it, then put double the quantity of chicken to celery into a basin, and mix in the following sauce; stir it in carefully so that all the strips get coated with it, then pile it high in some nice dish, and garnish the edge with the young green leaves of the celery.

Sauce.—Put two raw yolks of eggs into a basin, work them with a wooden spoon with a good pinch of salt and dry mustard; then slowly drop in two tablespoonfuls of salad oil, working it all the time; then add two tablespoonfuls of thick cream and a dessert spoonful of tarragon vinegar.

Grilled Ham with Mushrooms.—Lightly butter two baking tins. In one lay some thin slices of ham, sprinkle them with dry mustard, and cover with a buttered paper. In the other tin put some peeled mushrooms, season them with pepper and salt; cover these also with paper. Cook both in a hot oven for a few minutes and dish them on dry toast, putting ham and mushroom alternately. Sprinkle the whole with chopped parsley and serve as hot as possible.

Eggs with Piquant Sauce.—Butter a frying-pan, break the fresh eggs into it, and put it carefully on the stove. As soon as the whites begin to set place the pan in the oven; leave till the yolk is set but not hard, take it out, and cut out each egg with a plain round cutter; dish them on a warm dish and pour the following sauce over them: Sauce—Put two table spoonfuls of French vinegar in a little saucepan; when it boils add a dessert spoonful of thick gravy, warm them up together, then stir in by degrees one ounce of fresh butter, add a little pepper. Do not let the sauce boil. As soon as it is hot pour it over the eggs.

Cheese Fritters.—Make a good batter by mixing together two ounces of flour, the yolk of one egg, half a tablespoonful of salad oil, half a quarter of a pint of cold water. Whip the white of the egg very stiff with a pinch of salt, and stir this in at the last. Just before using the batter cut some small pieces of cheese about an inch and a half long and half an inch thick—Gruyère or Cheddar is the best. Dip these in the batter and fry in clean boiling fat till a nice golden colour. Take up to drain on kitchen paper and dish up on a dish paper. Sprinkle with chopped parsley and serve very hot.

### Trained Nursing in Sweden.

The chief training school for nurses in Sweden is at Sophiahemmet (Sophia's Home) in Stockholm, so called after the present Queen of Sweden, who founded it, and to whose continued personal interest and help its success is largely due. Having been herself a sufferer for many years, it was perhaps owing to this that her attention was drawn to the need for skilled nursing, and to the advantage of educated

Sophia Home for Nurses.

gentlewomen taking up this form of work. Hitherto, with the exception of the devoted nurses connected with the Deaconesses at Erste (a suburb of Stockholm), this ministry to suffering humanity had been mainly undertaken by the lower classes, without any very definite training.

A very modest beginning was made in 1884 in a flat only where three or four young ladies were the pioneers of a movement which soon spread after the initial surprise and opposition to what was wrongly regarded as derogatory and unsuitable occupation had been overcome. A second flat was soon added, and in 1889 the building known as Sophiahemmet was opened. The King and Queen personally con-

The Nurses' Sitting-room.

tributed 60,000 kronor (£2,000 circa), which was supplemented by the proceeds from an entertainment with tableaux, &c., arranged by the Crown Prince and Princess. It has been enlarged several times, and the Crown Princess has identified herself in particular with the nurses' home which adjoins the hospital, and where the comfort and wellbeing of the nurses and probationers is assured. The Queen often visits and takes a personal interest in all, often having one or other of the nurses in attendance on herself, and always giving away the certificates with a few well-chosen words.

Sophiahemmet is arranged as a paying hospital, capable of accommodating sixty patients, who pay at the rate of kr. 1.50 (about 1s. 7d.)—kr. 7 (7s. 6d.) per day, according to the room or ward occupied. All the arrangements are well carried out, and the airy corridors, with comfortable sofas and armchairs for convalescent patients, are a leading feature. A certain amount of accommodation is provided for patients' friends who wish to be on the spot. The hospital is surrounded with delightful grounds, which include a charming fir coppice.

The surgical work seems to be particularly good, and antiseptic principles are thoroughly understood and worked out.

The nurses' training is for two years, but the agreement is for three years, the time being divided between the Sophiahemmet and some of the State hospitals, where the ward

A Nurse at the Sophia Home.

sisters impart the training which they have themselves received in connection with Sophiahemmet. In these Government hospitals the ward sisters are responsible for the nursing to the doctor alone, under whose care the patients are, as there is no superintendent of nursing. The housekeeper, or house-mother (as she is aptly called) arranges all household matters. Hitherto this plan has been found to work well.

Applicants for admission as probationers have to send in to the lady superintendent of Sophiahemmet a certificate of character from a clergyman, of health from a doctor (involving a very thorough examination), and also an account of their previous life and conduct. A good deal of stress is laid on the motive for undertaking this work, as viewed from a religious rather than a professional standpoint. Candidates must be Protestants, give proofs of a good education, and be of ages 21-35. Probationers can be received for one year's training on payment of 50 kronor per month (about £33 per year). On admission each probationer has to come on trial for two months, paying 100 kronor, and, if considered suitable, is retained for three years, one and a half years serving as probationer-pupil, six months as probationer-nurse, and the last year as nurse. The salary during the third year is 250 kronor (about £13), which is raised to 300 if the nurse is kept on the permanent staff for hospital and private work. Of these there are 75, some scattered over Sweden, others working in Stockholm, but all animated by the same spirit and looking upon Sophiahemmet as their common home. There is a Pension and Sick Pay Fund, to which grateful patients often contribute, more especially as the nurses are not allowed to receive any gifts from those who have been under their care.

At the recent exhibition in Stockholm there were some interesting exhibits, especially the operating table and bed for heart cases, and a model room comfortably fitted up for a private patient.

The patients' fees seem very low, and also the nurses' salaries in consequence, but it must be borne in mind that Sweden is a poor country, and the adequate payment of skilled labour is still a matter of education—a consultant's fee, in an ordinary way being often only 5s. 6d. (at his own house).

### A Book and its Story.

### PLAIN LIVING: A BUSH IDYLL.

"Plain Living" * is a delightful presentment of pastoral home-life in a settler's family in the Australian bush. It will be read with much interest by all who have relatives in the new country, and who has not some dear one who has gone forth to seek his fortune—not always, alas! to find it—in that far-distant land? Mr. Boldrewood writes always as one who knows, and his long and varied experience of colonial life makes all that he writes of value to the reader. His writing has force and intelligence; a graphic, often poetic, power of expression, which gives life to the scenes and characters pourtrayed. His latest romance has all these characteristics, and gives a very vivid picture of the delights and disappointments of a squatter's life. "The life of a pastoral tenant of the Crown in Australia is, for the most part, free, pleasant, and devoid of the cares which assail so mordantly the heart of modern man in cities." Yes; but a few pages further on we come to the reverse of this soothing picture. "Striking exceptions to this rule are furnished periodically. 'A dry season,' in the bush vernacular, supervenes. In the drear months which follow 'the flower fadeth, the grass withereth,' as in the olden Pharaoh days, 'the waters are forgotten of the footstep'; the flocks and herds which in the years of plenty afford so liberal an income, so untrammelled an existence to their proprietor, are apt to perish if not renewed. Prudence and energy may serve to modify such a calamity; no human foresight can avert it. In such years a revengeful person would desire his worst enemy to be an Australian squatter. . . . He would mark him day by day, compelled to await the slow, advancing march of rain—hopeless, eradicable—which he was powerless alike to hasten or evade."

The story opens with Harold Stamford brought face to face with pecuniary difficulties arising from the above causes, difficulties which he is unable to avert. "And as he rode along on a favourite hackney, palpably low in condition, with bent head and corrugated brow, it needed but a little penetration to note that the iron had entered into his soul." And yet "God's mercy was above all. In it he would trust until the actual moment of doom. And still, as he marked the desolate dusty waste across which the melancholy flocks feebly paced, and as he saw on every side the carcases of animals that had succumbed to long remorseless famine; as he watched the red sun sinking below the hard unclouded sky, a sense of despair fell like lead upon his heart, and he groaned aloud."

Surely this is an outlook dark enough; but the clouds passed, or, rather, they "gathered overhead," which is what everyone was praying for, and the following extract from a letter shows the exuberant joy with which the falling skies were received: "We have had Rain! Yes, rain, in large letters. Forty-eight hours of steady rain! Five inches, sixty points. Didn't it come down . . . floods and waterspouts! The drought has broken up! The river is tearing down a banker. All bother about feed and water put away for another year at least. Hurrah! Hurrah! Hurrah!"

The breaking of the clouds brings blessings; better times dawn, and everything revives with characteristic colonial buoyancy. The story of the Stamford family, their devotion to each other; the chivalrous bearing of the men to their women folk; the loving, self-sacrificing services rendered in return; the charming, unaffected manners of the girls; the utter absence of slang or other up-to-date characteristics of the modern maid, makes very pleasant reading.

The whole setting of the scenes is so unlike and so far removed from anything in a present-day household at home that one must go back to the characters of Mrs. Gaskell or Jane Austen to find girls like these charming, cultivated, domesticated, Australian heroines.

As a set-off to the free, breezy atmosphere of this up-country home we are introduced to the family of the rich, financially successful cousin in Sydney. The Grandisons are a specimen of the type found easily enough in our own country. Rapidly acquired wealth and easy ascent of the social ladder in consequence, has not brought refinement or "gentleness" in its train. There is the same florid display in their pleasures and palaces there, as here; the same ceaseless pursuit of unsatisfied ambition; the disappointed, over-indulgent father; the scheming, worldly mother; and the selfish, indifferent children.

It is not with them, however, that the interest of the story centres, and one turns to the development of the Stamford fortunes with renewed zest after the glimpse of life and society in the gay metropolis of Sydney which the Grandison introduction affords. There is not a dull page in the book. Some readers may perhaps object to the mode of expression adopted by some of the characters as being a little precise, and even stilted, to the modern ear. It is certainly quite unlike our own free and easy style; but there is this to be said for it, in this very precision there lies a dainty charm, a simple sincere dignity lamentably absent from present modes—modes characterised chiefly by haste and flippancy.

No one, we think, can rise from the perusal of "Plain Living" without the feeling of refreshment we have spoken of, and we cordially recommend it to the notice of those who appreciate a wholesome, healthy-toned story.

* "Plain Living: A Bush Idyll." By Ralph Boldrewood. (MacMillan and Co. 6s.)

### Death in Our Ranks.

It is with deep regret we have to record the death, on Wednesday, the 23rd inst., at Dennistown, Glasgow, of Nurse McAllister, for thirteen years a most highly valued nurse of the Royal Scottish Nursing Institution, Edinburgh. She was beloved and respected not only as a most excellent nurse but as a beloved friend, both by her fellow nurses and her patients.

### Cremation.

By LADY WATKIN WILLIAMS.

THE sight of that tangled overgrowth of gravestones, Brompton Cemetery, at the recent funeral of the lamented William Terriss, must have stirred many thoughtful minds to the question of cremation versus burial. Brompton Cemetery is only one among many such nightmares of the grave, only one among many painful examples of the peaceful churchyard of hallowed associations becoming the charnel house of peril to the living; the teeming, unsightly, unrestful last home of the dead. More than three hundred years have passed since Sir Thomas Browne, of gentle, cultured memory, recognised the claims of cremation, and illustrated those claims by many an ancient example as well as precept; more than twenty years have passed since Sir Henry Thompson lent all the weight of his name and fame to the plea for revival of the custom. But long use and sentiment still hold their own against it, combined with that still stronger force, that time-worn, self-deluding prejudice, that oft-quoted, mis-quoted joy of bigots and despair of thinking men, that so inaccurately called "religious principle"; and together they successfully hinder the conversion of the many to the growing practice of the few.

For those who cling to sentiment let no harsh word be said, for all will sympathise in their love for the country churchyard, where sleep the quiet dead; in their reverence for all the memories that cluster about the grassy graves in "God's acre." Could daisies and yew trees still maintain their place wherever the dead should lie; could churchyards not give way to cemeteries and the overcrowding of both not give rise to conditions of danger and death, the cremation crusade need never have started. But it is not by sentiment that its onward march will be long arrested; sentiment is not bound by the iron fetters of prejudice, and even if it may not be persuaded of the symbolic beauty and poetry of "cleansing fires" with their ascending witness, its pain may surely be soothed by details which shall still associate death with calm and rest; for there is no reason why the crematorium might not find its centre in a "garden of sleep," peaceful as any memory-haunted churchyard of the past. The real difficulty is with those who cling to burial with all the tenacity of ill-founded religious prejudice, and a very superficial glance at facts will show on what frail foundations they rest their religious convictions.

The origin of Christian prejudice against cremation was clearly its previous adoption among the heathen nations of antiquity, although, as has been pointed out by Sir Thomas Browne, the question was disputed even in those old days before the advent of Christianity, Heraclitus, for instance, advocating burning, while Thales and Hippon argued the claims of burial. But cremation was undoubtedly the universal practice among all ancient peoples except the Egyptians, Jews, and Chinese. Should, however, Egyptian, Chinaman, or Jew seem to have a stronger claim on our imitation than Roman, Greek, or Celt, there were yet reasons for their special customs which tend to make their example inoperative on us. The Egyptian, for instance, not only regarded the body itself as sacred and as literally destined for immortality, but Herodotus stated that they regarded burning with horror, because to them fire was a savage monster, devouring all it can lay hands on, and when satisfied, dying together with its prey. Being also forbidden by their laws to suffer any animal to live upon a dead body, they were driven to embalming as a protection against worms.

The Chinese had no prejudice as to preservation of the body (and, indeed, had but vague ideas as to the immortality of the soul); but they, and probably the Jews also, were precluded by one reason at least from any idea of cremation—want of fuel, so they might naturally resort to earth burial as the simplest and least costly mode of sepulture. The Jews may be thought to have a special claim of authority over Gentiles who revere their history and accept their Scriptures, but what is the argument as regards the Jews? Their country provided no fuel, so cremation was not suggested. It possessed wide wastes of rocky, uncultivated land; it was but sparsely populated, so that burial in caves or sepulchres would in such a case seem but the natural outcome of the affections of a wealthy people in the earlier stages of development. They could at least have had no religious objection to cremation, for at least two instances are given in their sacred and secular history where burning of the dead was ordered and carried out without remark or remonstrance. Also Moses, who was so minute in directions on all points of moral and religious law, never legislated on the question at all; while St. Paul, Jew and Christian in one, who must have known of the custom so long in vogue in Greece and Rome, was equally silent on the subject to his converts. In fact no specific prohibition is given in either the Old or New Testaments, and the founder of Christianity, in spite of the importance of the fact, did but follow the immemorial historical custom, and neither established a precedent or inaugurated a new idea when He was laid in a rock-hewn chamber or sepulchre. The indifference of the early converts to the destruction of their bodies is a proof that to them cremation could have no terror, as regards immortality of the soul; and in truth cremation should never have become a religious question at all, its origin was purely sanitary, as the claims for its revival are purely sanitary now.

The early Christians abjured its practise, not from "religious principle" but because in the fervour of their new faith they abhorred all that was associated with their former heathen condition. It was left for later ages to turn this natural sentiment of reaction into so-called religious motive and to make a fetish of corruption and the grave. In consequence our overcrowded churchyards and teeming cemeteries, with their probable malarial fevers of the future, form a sorry legacy to our descendants, in marked contrast to that bequeathed to us by our own island forefathers, who burned their dead and then interred their remains in urns. "As cannot but appear," says Borlase, in his "Antiquities of Cornwall," "from the mixed number of barrows and urns found everywhere, and from ashes mingled with the earth." But prejudice dies hard, and although chapter and verse could be given to prove that towns have been decimated by plagues after the opening up of old plague pits; by fevers after the opening up of old burying-grounds; although there is no real moral or religious objection to cremation, and although it is admitted that the few practical difficulties in the way of its general adoption can be overcome, it is to be feared that unless those who understand the importance and the morality of the subject will lend their persuasion and argument to the cause, it will be long before the many will be won over to sparing the living by a simple change of funeral rites for the dead; long before they recognise that their cherished "religious principle" is but a mere excrescence on the convert's "natural prejudice" of more than eighteen hundred years ago.

### Minor Appointment.

STOCKTON AND THORNABY HOSPITAL. — Miss Barbara Anderson, who has just finished her three years' course at Beckett Hospital, Barnsley, was appointed Charge Nurse of this hospital on April 1st.

### Miss Lilias Hamilton, M.D., at the Royal British Nurses' Association.

THE Royal British Nurses' Association had the good fortune to secure the Ameer of Afghanistan's lady doctor to lecture upon her unique experiences in the East. The room of the Medical Society of London, at 11, Chandos Street, Cavendish Square, was well filled on the evening of March 25th, in spite of the inclement weather. Miss Wedgewood, Matron of the Royal Free—Miss Hamilton's training school—took the chair.

Miss Hamilton covered so many points in her address, all so full of information, that it would have been fairer to her, and possibly still more interesting to her hearers, had she given a series of lectures instead of one. Recognising the impossibility of still further condensing her subject, "Afghanistan," we shall merely take a few points more directly connected with her medical enterprise. Miss Hamilton describes the Ameer, whom for four years she served so successfully, as a "magnificent savage, capable of the most chivalrous courtesy and of the most unnecessary and barbarous cruelty. Few Orientals understand anything of his subtle and capricious nature, no Western can ever hope to do so." The country over which he reigns, the wild and conflicting tribes which he has welded into a united kingdom, are as full of contradictions. It is the land of "blood and stones," "of sunshine." "It is India exaggerated." "It is fertile and rich beyond conception in fruit and flowers." "It is a country of barren peaks and snow." Each and all of these descriptions is absolutely true. Amongst the lantern slides that brought the scenes before the audience was one of a recent palace of the Ameers. The house was at some distance and the foreground was a luxuriant vineyard. A few years ago this fertile tract had been a characteristically Afghan waste covered with huge stones amongst which scrubby bushes grew. The stones removed, irrigated with water brought in aqueducts a distance of twelve miles, the wealth of the soil and climate converted the waste into a garden.

The people resemble their country. Numbered amongst them are to be found the most handsome, cultivated, courteous, tenderly human, in the true sense of the word, gentlefolk, and the most hideously repulsive, degraded, and cruel of mankind.

For two years Dr. Lilias Hamilton was in close personal attendance on the Ameer and saw the outside world very much through his spectacles, and for the next two years of her residence, being released in great measure from the Royal service, she opened a dispensary and found that the people had also a very fair case on their side. In Afghanistan, however, no one speaks of such things; it is dangerous. This instance illustrates the state of affairs very well.

A high official visited Dr. Hamilton one evening so obviously depressed that she enquired the cause. He then reluctantly told her that a bright, handsome page-boy had offended the Ameer, who had ordered him to be severely beaten and afterwards put out into the snow, then three feet deep, to die. "I must go to the Ameer," exclaimed the Englishwoman at once. "Most probably your interference will only add another torture to the lad's sufferings," replied the official. "What can be done? Can you do nothing?" "I must try." "And if you don't succeed?" "The Ameer will probably have my tongue cut out." After an hour, however, he returned jubilant. The Ameer had consented to end the punishment after the lapse of another hour. The hour passed slowly, Dr. Hamilton watching the clock with sick despair. Then the boy was brought in for her to revive. He is alive, and now holding an important post under the Ameer.

The dispensary was largely attended; 350 was a normal day, and sometimes the number reached 720. The dispensary operating theatre, and consulting-room was the garden. The patients as they arrived were roughly sorted by her guards and sent to different corners of the garden. Dr. Hamilton went round, wrote her prescriptions, and the nurse, Mrs. Daly (a member of the R.B.N.A.), and the compounder administered treatment. Operations for cataract were frequent, and ophthalmia was so very bad that it only yielded to heroic measures.

Before Dr. Hamilton left she instituted calf vaccination stations and instructed eight stalwart men how to prepare the lymph. In order to discover some groundwork on which she might plant this new knowledge she requested instruction in their system of physiology. "What, for instance, becomes of the food you eat?" she asked. They replied, "After it is masticated it goes into the stomach and is divided into two parts—good food, called chyle, and waste. The chyle goes to the liver, which is a great fire, where it is divided into four: First, air, which mounts to the brain and causes delirium and madness; second, gas, which causes wind and tumours; third, slime, which causes dysentery and boils; and, fourth, good food, which passes straight to the heart. The heart is also a fire, and divides the food into two parts, the one sustains the soul, and the other nourishes the tissues." "But you have other organs. For instance, what is in the chest?" "Oh, the lungs, of course; they are the punkahs that fan the heart."

A unanimous and cordial vote of thanks was accorded to the lecturer, and afterwards most of those present examined with interest a piece of white silk embroidery on cashmere wrought by the women of Cabul. As such work rarely leaves Afghanistan this is probably the first exhibition of its kind in London.

Nurse Daly is still busy in Cabul, carrying on the work single-handed, but as accidents are frequent she must long for a surgeon's aid as well as the moral support of one of her own race.

### The Nursing Sisters of St. John the Divine.

THE Nursing Sisters of St. John the Divine is an old-established nursing sisterhood of the Church of England, and the methods and aims of those ruling it are conservative. Pains are taken to cultivate the moral, as well as the mental and manual, qualities of the probationers undergoing training; and afterwards to attach them to the home. The superior officers are ladies; for, although respectable women of all classes are acceptable as nurses, it has been found that the influence of gentlewomen in training gives a certain refinement and a standard of good manners that is not to be obtained without it. The ages of candidates must be from 24 to 35. For the whole term of the three years' training each probationer receives the sum of £10, with board, lodging, and uniform. The wages are then increased annually until they reach £35. A comfortable home is provided for nurses when not at work and in their old age. If a nurse chooses to leave the association at the end of any stipulated period she is at liberty to do so, and the Sister Superior gladly acts as a reference. This is considered by some to be more valuable than a certificate in many ways. For instance, it by no means follows that after a nurse has quitted her training school and knocked about the world for some time she remains a desirable attendant in sickness; but if she is so, and belongs to the Nursing Sisters of St John, the Sister Superior's recommendation is an up-to-date certification of the fact. Perhaps this is the reason that the nurses in connection with this home are held in great repute by those who have employed them.

### Promotion.

By One Who has been Promoted.

Speaking generally, it may be taken for granted that a satisfactory probationer will make a successful staff nurse; it is not so certain that every efficient staff nurse would make a capable sister, and it is open to discussion whether the most competent ward sister is likely to become the best matron. Let us state our hypothesis.

Taking the hospital as the mother country, each ward may be likened to a separate colony with its own system of self-government. The sister is its supreme head. The whole working of the ward is under her control; she formulates, directs, regulates, and dominates everybody and everything coming under her immediate jurisdiction. By her the patients are kept in order, the probationers are trained, the staff nurses are instructed, the dressers are admonished, the doctors are satisfied, the work is harmonized. According to her rule either efficiency is maintained and happiness promoted, or incompetency reigns and integrity is lacking. Unconsciously, as time adds to her experience, she is liable to become more and more autocratic, until at last she may exclaim with Louis XIV., "*L'Etat c'est moi*"—the ward! It is I. The ancient king or Könning (Can-ning) was the able man, chosen out by reason of his strength and power—the ward sister is (or ought to be) the able woman elected because of conspicuous merit. Those whom she is sent to command believe her to be the wisest, the fittest, the best. They see her clothed with the dignity of her office; they must to a greater or lesser degree be willing to allow that "the king can do no wrong." Unless the autocrat become a despot this is not in itself an evil state of affairs, but is it the best preparation for what may follow?

There generally comes a time when, for one reason or another, the cherished ward must be relinquished, promotion must be sought for or accepted, and an iconoclasm is inevitable. In one brief moment the treasured sister must pass from the warm, adulatory atmosphere of her small but obsequious realm into the frigid zone of her new domain. There the quondam autocrat finds she must accustom herself to the trammels of a limited monarchy; the sovereign power of the matron must ever be subject to the constitution, and she may sometimes find it difficult to agree with her parliament. A vista of trouble will open itself to the unaccustomed tyro, she will see looming before her a void which cannot be filled—for she knows the new hospital can never be to her as her own ward. The work, too, will seem so inferior; servants will be well in the foreground, and the nurses will appear to have retired from her direct personal influence.

Beef and mutton and suet puddings monopolize her attention instead of the absorbing interests of pathology; giving out stores substitutes the art of "doing dressings." No one adulates her, no one seems to have any special confidence in her—so great is the metamorphosis, she feels no confidence in herself. And as the weary days drag on devoid of interest, she finds herself yearning for her own little colony, and wondering what induced her to leave its shores.

Such, we contend, must be the inevitable experience of every valued ward sister who steps on to the higher platform, and her success will be exactly measured by her capacity for adapting herself to her altered circumstances. The bare suggestion at first that the dreary wilderness of thorns and stings may eventually blossom into a happy home would be repudiated with derision; nevertheless, it may console such an one to know that parallel cases have occurred, and that history has a knack of repeating itself.

### A Letter from Poona.

### PLAGUE CASES.

By a Sister on Duty.

As plague has disappeared from my district, at least for the present, I have been transferred to my original post. About three weeks after I had left the camp at Kirkee I was very much distressed to hear of the death of a little lad of five years old, who had seemed to be doing so well when I came away. An elder brother had died out of hospital, the mother two days after admission, and this little fellow had been in the hospital for nearly three months. He had four buboes, one on either side of the neck and one in either groin; also two sympathetic swellings, one just above the knee on the right leg, and the other in the calf of the left leg.

The bubo in the right groin and also that on the right side of the neck were opened, and I have seldom known so much discharge or for it to continue so long a time. Of course the child grew very weak, but after the sixth week began to improve rapidly. It was quite an event when wrapped up in blankets he sat up in bed for the first time, and before I left the camp we could carry him about. Imagine then my disappointment when, after having been away about a fortnight, I heard that he was refusing all nourishment and could hardly be roused. Within the next week he died. His temperature for the first three weeks ranged from 101 to 104 deg., nothing seemed to bring it down, even for a time; his screams were incessant and piercing, exactly like those in meningitis, as was also the restlessness of the head. Very gradually this stage wore away, to be followed by that of excessive weakness and drowsiness. The temperature was normal and sub-normal for the last three weeks. For the first three weeks ice had been kept on his head day and night, and both swelling and bubos were painted with belladonna. As frequently happens, the child showed a tendency to bronchitis, but a cotton wool jacket soon rectified that. He was also subject to diarrhœa throughout his illness, but his pulse was always fairly good. We had two specially marked cases of deep seated buboes, one of which occurred in the old camp. A young woman was brought in to be placed under observation, with very high fever and delirium, but not the most careful examination discovered any inflamed gland. It took two attendants all their time to keep her on her cot, and she was one of the most noisy patients. Her temperature never fell below 103 deg. during the first eight days, and it was by no means easy to feed her. On the fourth morning, however, a distinct swelling of the left upper half of the chest and deep down in the axilla was noticed, a gland could just be felt, so she was moved in the plague chuppers. The bubo became very large and was eventually opened. The highest temperature registered for the week before the incision was 100 deg., and consciousness returned. She made a splendid recovery.

A very sad case was that of a man employed in the search party. At first sight it was apparent that his was a plague case. There was the restlessness, groaning, abject terror, congested eyes, and foul tongue. Upon unfastening his coat to examine him a distinct swelling of the entire left half of the chest was discovered, and deep down in the axilla there was the bubo. There was no need to ask whether there was any pain; how the poor man had managed to keep his arm by his side in his endeavour to hide it was incomprehensible. It was so deep-seated that it seemed that it must be actually on the ribs. His condition was serious; he was barely conscious, his pulse very feeble though high, and his temperature 102 deg. The vomiting and diarrhœa were incessant. The next day, though his temperature never rose higher than 103, all his bad symptoms increased in intensity, and he broke out into clammy sweats. His restlessness and distress of mind were painful to witness. On the third morning another bubo appeared on the left side of the neck, and he became much more conscious, kept his food down much better, and in the early part of the morning his pulse improved, but respiration grew hurried and shallow, expectoration became frequent and thick. Death took place very suddenly that afternoon.

We had one very curious case, in which the tongue on admission was quite clean, but daily became a little more characteristic, until it was a typical plague tongue. The patient was a girl of eight years old; the bubo, an enormous one, in the right groin, and was one of the few which did not suppurate. It was intensely painful, and very hot to the touch. The highest temperature registered 103 deg., but the child never became delirious. Her's was a very long case, indeed. As a rule the majority of buboes suppurated, and then the patients appeared to do so much better, the convalescence being much less tedious. The attendants and the patients themselves asked daily when the incision could be made, and were most unhappy if told that the bubo was subsiding.

The friends and children who were admitted with the patients—the former as attendants, the latter often of necessity, there being no one to take care of them—gave a great deal of extra work; but, on the whole, they behaved splendidly, and were a real help, being so willing to help the friendless ones in their own chuppers and sinking caste distinctions to an astonishing degree.

There were often two relations of the patients in attendance on a case, the only drawback to this arrangement being that at first they often gave the patients solid food (some of their own rations) with disastrous results; but as I began to gain my footing and be known well amongst them this was of rare occurrence, though a native has a rooted objection to milk diet, being by no means considered food by them.

The buboes were often covered with large blisters when the patients were admitted, owing to the habit of rubbing them with the scrapings of the marking ink nut. The result was agonising, but they have unbounded faith in it. It is applied to any ordinary swelling as a matter of course. Applying leeches is also a favourite native remedy, the number employed apparently being immaterial.

### Mediæval Practice in the Care of the Sick.

### VI.—"LADIES," "WISE WOMEN," AND "WHITE WITCHES."

In the Middle Ages nuns had by no means the monopoly of nursing; a very large proportion of surgical nursing was performed by secular ladies, who tended wounded soldiers within their own bowers, or in mediæval castles, and a "Dietoric" exists which was written about 1430, containing simple rules for the guidance of any "if so be yat leches doon yee faile." Rather a frequent occurrence probably, for chatelaines and their bower ladies were nurses and doctors both in those turbulent days. A third class of nurses were the "wise women," or "white witches," learned in herbs, having charms for any ill to which village life was liable, from childless marriages, "overlooked" cattle, to corns or warts, and who, by the exercise of a little common sense, combined with a really experienced knowledge of the nature of plants, frequently succeeded in curing ailments which defied the skill of the learned faculty, getting themselves not seldom into trouble on account of such charity. Thus, "while the men who professed the healing art were generally astrologers and alchymists, lost in dreams of the elixir vitæ, the philosopher's stone, and such mummeries and quackeries as made them favourite subjects for comedy and satire," these three classes of women "were accumulating a vast fund of practical and traditional knowledge in the treatment of disease, and in the use of various remedies."

Having seen, if somewhat dimly, the character of nurses, we may proceed in endeavour to form some idea of their methods and the nature of the treatment to which they subjected their patients.

Malory tells us that the "maiden Tinet came to Sir Beaumaris and unarmed him, and searched his wounds, and stinted his blood," after a fight, and the same lady on another occasion bade him be of good cheer, "for I undertake within these fifteen days for to make you as whole and lusty as ever ye were. And then she laid an ointment and a salve to him as it pleased her." La Belle Isond was a "noble surgeon"; she "searched" Sir Tristram, "and found in the bottom of his wound that therein was poison, and so she healed him," and Tristram "cast great love" to her.

In the Carlovingian romance of "Gaufry," when Robastre is given up to die from the effects of his wounds, the "good wife" of the traitor Grison undertakes to cure him, and accordingly we read: "And she went to a coffer and opened it, took out of it a herb which has so great virtue that whoever takes it will be relieved from all harm. She pounded and mixed it in a mortar, and then came to Robastre and gave it him. It had no sooner passed his throat than he was sound as an apple." The magic herb sounds a little like a reminiscence of the recipes Helen of Troy obtained in Egypt; but medicinal herbs were grown in every English garden, and in Moreton Old Hall, Cheshire (a most interesting architectural survival of Tudor times), may still be seen a wondrous chest, with dozens of little drawers made to contain the dry herbs. With what amount of truth we know not, it is said that the English were not such good herbalists as some other nations, however, and that Catherine of Aragon imported some of her vegetable remedies from Holland. Scott's Rebecca has many prototypes; in the "Fierbras" romance a Saracen princess "had in her chamber the powerful mandiglore (mandrake), which she applied to the wounds of Oliver, and they were instantly healed." But though the Saracen knowledge may have been superior, we had gardeners in England who wrote upon the effects of the plants they grew in their monastic gardens. In the fifteenth century MSS., now in the British Museum, we find a list of plants considered necessary for a garden, and the list is classified thus: "Of the same herbes for pottage—borage, dandelyoun, mynt," &c. "Of the same for sauce," and later, "also herben to stylle," "endyve, red rose, rosemary, dragons, . . . wermodle . . . wylde tansey, sauge, isope," &c. We know the names of two monks who were famous gardeners in their day—Nicholas Bollarde, of Westminster, and one Godfrey Palladu, who wrote upon his craft, while tradition says the Æthling Princess Matilda was saved from an uncomfortable marriage with the Red King by a ruse of her rose-growing aunt, the Abbess of Romsey.

The uses to which home-grown herbs were applied may be seen from such recipes as the following: "Against headache"—"Take a vessel full of leaves of the green rue, and a spoon full of mustard seed, rub together, add the white of an egg, a spoonful, that the salve may be thick, smear with a feather." For sore eyes—"A noble craft! Take equal quantities of balsam and virgin honey, mix together and smear with that." Fennel, southernwood, celandine, "burnt salt" mixed with honey, green coreander, &c., were constantly used in ophthalmic nursing, and a sovereign remedy for bad eyes recommended for centuries was human milk. "For every hard tumour or swelling dry beans and seethe them without salt, then mingle with honey and lay on." A leprous body was treated with dock and silver weed, pounded and boiled in butter with a little salt, "wound salves" were composed of many herbs, honey, wine, or oil, &c., and were frequently boiled in butter, smeared on a cloth, and applied. The foregoing recipes will be found with numberless others in a curious book printed in the Rolls series, "Leechcraft, starcraft, and wort cunning," &c., but monastic scribes jotted down anywhere a good "cure" they chanced to hear, to aid their memories and serve their brethren; for example, "For to destroy a wrong nayle, otherwise called a Corne. Take wylde tansey, grynd yt make yt mashe and lay it thereto, and it wyll bring yt owght," is a useful piece of information, found, sandwiched between moral sayings, proverbs, and other matter, in a MS. printed by the Early English Text Society, Vol. 15

### The Book World for Women and Nurses.

[We invite Correspondence, Criticism, Enquiries, and Notes on Books likely to interest Women and Nurses. Address, Editor, The Hospital (Nurses' Book World), 28 & 29, Southampton Street, Strand, London, W.C.]

Perfect Womanhood: A Story of the Times. Frederick Gant, F.R.C.S. (Digby Long and Co.)

It is evident that the author of this volume has not intended so much to write a novel with a formal plot, as to draw a series of portraits for the purpose of illustrating an ideal; and in the gallery through which he invites us to wander there are many pictures before which one has pleasure in pausing. Of these pictures it must suffice to say that they are sketches of various types of womanhood, of whose loves and joys and sorrows we catch a glimpse, or rather something more than a glimpse, for Mr. Gant's touch is firm, and his method affords ample scope for the delineation of character. In reading the pages we are reminded of Wordsworth's lines:

"A perfect woman, nobly planned,
To warn, to comfort, and command;
And yet a spirit, still and bright,
With something of angelic light."

These lines may be taken as the epitome of the character of Mr. Gant's heroines. Each has set before her an ideal which she strives to reach. His theory is that "modern womanhood in its highest development is essentially the impersonation of the spiritual and intellectual (often blended) powers of feminine humanity," and in support of this theory he has brought upon the stage these heroines of his, the description of whose noble lives and lofty ideals imparts an elevating tone to the book. The character in "Perfect Womanhood" whom we follow with most interest is that of Sister Eve, a character of uncommon loftiness of purpose, a veritable example, indeed, of one type of perfect womanhood, of whom it is good to read.

The Register of Trained Nurses of the Royal British Nurses' Association for 1898. (Offices, 19, Old Cavendish Street, London, W.)

The Royal British Nursing Association has just published a list of the nurses who are members. It is hardly so well got up as might reasonably be expected for the 2s. 6d. charged for it, the appearance and paper being distinctly inferior. We also notice that in some instances the name of the training school is omitted. In addition to the register the book contains a preface setting forth the causes that led to the formation of the association and the regulations to be complied with before a nurse can become a member.

The Zenana; or Woman's Work in India. Vol IV. (S. W. Partridge and Co. Price 2s. 6d.)

Women's work in India must perforce be carried on in the zenanas. The missionary societies, in their endeavours to reach the women and carry to them the Gospel of Christ, soon found that their ignorance and physical sufferings cried out for education and healing almost as much as their souls for spiritual enlightenment. How much has been accomplished let the unostentatious volume of the "Zenana" tell. "There are now twelve women's missionary societies in Great Britain and Ireland, supporting 770 missionaries, of whom 38 are medical, in various foreign fields. They employ 2,000 native women, and have upwards of 60,000 girls in their schools." Satisfactory as these numbers are when looked at by themselves, or in comparison with what has already been done, the thought that there are 140 odd millions of India's women yet living in the seclusion of the zenanas adds tremendous force to the appeal for more money to increase the work. The missionaries helped greatly during the recent famine, and England gave money generously; but there is still a famine which calls for healing in sickness, skilled nurses, and the unselfish devotion of the ministry of Christian women. The "Zenana" contains much interesting information, it is clearly printed on nice paper, and prettily illustrated. The frontispiece represents two of the native nurses in "Lady Kinnard's Hospital," Lucknow.

### Everybody's Opinion.

[Correspondence on all subjects is invited, but we cannot in any way be responsible for the opinions expressed by our correspondents. No communication can be entertained if the name and address of the correspondent is not given, as a guarantee of good faith but not necessarily for publication, or unless one side of the paper only is written on.]

### HOW TO PEPTONISE MILK.

"Helpful" writes: In last week's Hospital "Puzzled" inquires how to peptonise milk? I should like to help her if possible from my eight years' experience. The following is, I believe, the simplest and best way. Be sure that everything used is absolutely clean and the milk quite fresh. Take a pint of milk and put half of it in a saucepan on the fire and let it just come to the boil (without boiling), then pour back on to the cold half pint. Mix half a tube of zymine powder (Fairchild's) in a teacup of warm water and stir it into the milk, pour all together into the saucepan, cover, and leave in warm place to digest for ten minutes, then boil up, and pour into the jug. Care must be taken not to put the milk on too fierce a fire, as it is more apt to separate; if the separation takes place, and the milk appear curdled, pouring backwards and forwards between jug to saucepan will often put it right again.

### THE CHILDREN'S NURSE.

M. E. Smith writes: I see by The Hospital of last week that the Norland Home is the only recognised home for training children's nurses. I do not know Norland Home, but I do know of several homes where infants are received and where nurses should find the necessary training in the care of very young children. Unfortunately we are unable to receive ladies for want of room, but we do receive a limited number of superior class women who are willing and able to take an equal share in the cleaning of the nurseries as well as the children. Two years in a home of this description should fit them to receive the highest salaries given. It is very plain that the training of children's nurses is a known and felt want in the country. It should be no waste of time for those wishing to become sick nurses who are yet too young to make it desirable for their own health to enter the sick ward of a hospital, to train at such homes as I have mentioned. I need hardly say that with a number of infants there is very real sick nursing to be done from time to time.

### GOOD MANNERS.

"A Lady Superintendent" writes: In these days one is constantly hearing complaints of nurses' manners. Why is it? Mothers will nurse their own children through long illnesses rather than have a trained nurse in the house. Surely this ought not to be? Why cannot one and all try rather to gain the respect of the public by being at least civil and better mannered? I am a gentlewoman, having trained at one of the largest London hospitals; have acted "sister" at two, and superintendent of a good-sized provincial hospital. I wish now to relate my experience of the past few weeks. I have had occasion to go to the out-patient department of three of the London general hospitals; at two I received the greatest politeness, both from the sister, nurses, and even porters. At the third I was met by a nurse, about thirty, who treated me with the utmost contempt; her manner, toss of head, &c., was far too horrid to be imagined. She saw by my uniform that I was a nurse, so that she might have greeted me somewhat differently, if she is accustomed to treat the poor patients so. The private nurse is the one generally complained of, so that I was most surprised to find a nurse with such airs on the hospital staff.

How can such a woman teach probationers, if she is bereft of common civility? Is it want of thought or good breeding? Can anyone suggest some remedy for this kind of thing, for the lady superintendent gets blamed, not the nurse? I should add that the house surgeon who replied to my inquiries was most courteous and polite; in a moment I recognised him as a gentleman. I am told that the nurses at this hospital are ladies. Surely, not the one who addressed me.

### ROYAL NATIONAL PENSION FUND FOR NURSES.

"AN UNSATISFIED POLICY HOLDER" writes: May I, through the columns of your paper, ask a question about the Royal National Pension Fund for Nurses? What are the benefits compared with other annuities? A nurse invests all her hard-worked earnings in the Pension Fund, and at the specified age when her pension becomes due, she receives the same, but with no more advantage than she would get in any other insurance, for most insurance offices give bonuses. Then if she die, say, a few months after the pension is due, the whole of her earnings goes to the Fund; thus, if she may have poor relations, they receive no benefit, whereas if she invested her money, say, in safe mortgages, &c., she would be getting the interest all the time, and at her death the capital would benefit some of her own relatives. Surely this would be a less selfish way of investing her money. May I ask what the special benefits of the Pension Fund are? Of course, we know it is safe; but so is the Post Office, insurance, and many others. Any information I shall be grateful for.

*** There is great difficulty in explaining anything to anyone who writes so positively on a subject about which she is so ignorant. She says that "when her pension becomes due she receives the same" (whatever that may mean) "but with no more advantage," &c. We shall be glad if the lady would tell us what insurance company gives bonuses on annuities. We have never heard of one. If she wishes her heirs to receive back the balance between what she has paid into the Pension Fund and what has been paid to her in pension up to the time of her death, she must also be prepared not to receive anything further in pension should she live on after she has been repaid the amount of the premiums which she has contributed. Obviously no annuity business can be carried on except by averaging short and long lived persons. The Pension Fund is not established for the sake of the "poor relations." If the nurse intends providing for them, certainly an annuity on her own life is not the way to do it.

### NURSING IN WEST AFRICA.

"A NURSING SISTER FROM WEST AFRICA" writes: Reading in THE HOSPITAL of March 12th Miss Kingsley's lecture at the Royal British Nurses' Association, I see she gives her views on nursing in West Africa. Some of her remarks may be misleading to those who do not know West Africa, at least, so they seem to me. For instance, she gives the idea that the English have no hospitals out there. Such is not the case; there are certainly two Government hospitals with English nurses—one at Accra and one at Lagos. Then again, she thinks that "a staff of hospital ships lying off the coast would be a practical way in which to deal with the nursing question." They might be very useful were it not for the surf. Anyone who has seen a bad surf—and it generally is a bad surf—would not care to go across it unless they were obliged, and to a sick person it would be very bad. Sometimes the boats are turned over; other times they get into the middle of the surf and there stick, not able to go towards the ship or back to shore. What would become of the poor patients then? A man strong and well will not go through the surf unless he is obliged for fear of getting wet, which generally means getting fever. English nurses have been at Accra now for more than a year. Two went out in February and one in May, 1897. Of the two, one—the matron—was taken ill on June 15th, and the other on the 16th. The matron was ill over five weeks, and was then invalided home for good; the other was ill for some time, and then went with the matron as far as Las Palmas. The nurse who went out in May was on the sick list eleven days only, but she had over four months to work and live alone. The hospital at Accra is a very good one; there are three wards and a sitting-room for Europeans. Of course, anyone coming straight from England might think them bare, but no doubt by degrees they will be better furnished. The nurses do eight months' work and four months' leave; the salary is high if compared with salaries in England, but so is the "death-rate."

### A NURSE'S AMBITIONS.

"C. W." writes: I am not a nurse yet, but I hope soon to enter into a course of hospital training, and when I do this will be my aim in life: To be of some use in the world, not to strive to become a matron, or to be the owner of a nursing home, but to soothe not only the wounded flesh, but also the troubled spirit, to be a comfort to them all, to deny myself, to spare no pains, trouble, nor time that shall help or cheer any one in pain or loneliness, to think not of self, nor of how great and grand I may become some day, but to give all my thoughts, talents, and love to those suffering ones entrusted to my care, content to fill a little space in life, if only I may be of some comfort to my weary brothers and sisters in the sick world, to be a bright companion to all; in fact, to spend my life for the suffering ones around me. And then, when my nursing days are over, if I can look back and see that I have been a help and a comfort to some, even though I myself should be quite forgotten, I shall be abundantly satisfied. I cannot say why some nurses succeed while others fail, unless some put their head and hands in the work and not their hearts. Perhaps some older than I can answer that question?

*** "C. W.'s" ambition should lie at the root of all Christian lives. But in order that she may succeed in carrying out her aims she must put more definite purposes before her.—ED. *T. H.*

### Notes and Queries.

**The contents of the Editor's Letter-box have now reached such unwieldy proportions that it has become necessary to establish a hard and fast rule regarding Answers to Correspondents. In future, all questions requiring replies will continue to be answered in this column without any fee. If an answer is required by letter, a fee of half-a-crown must be enclosed with the note containing the enquiry. We are always pleased to help our numerous correspondents to the fullest extent, and we can trust them to sympathise in the overwhelming amount of writing which makes the new rules a necessity. Every communication must be accompanied by the writer's name and address, otherwise it will receive no attention.**

*Slight Paralysis*

(1) Can anyone tell Nurse Squire (18, York Street, Baker Street) of a kind home for an old man who has had a slight attack of paralysis? A small weekly sum can be paid.

British Home for Incurables, Crown Lane, Streatham Common. St. Peter's Home and Sisterhood, Mortimer Road, Kilburn; 8s. a week. Brabazon Home of Comfort, Reigate; 10s. a week.

*How to Start a District Nurse.*

(2) It is very kind of Lady Superintendent to send us particulars of her experience on this subject. If we can learn anything as to the reason of the institution's action we will communicate with her upon the subject.—ED. *T. H.*

*The Hospitals of the Continent.*

(3) Can you tell me the name and price of a book giving a graphic and detailed account of the important hospitals on the Continent?—*Vera.*

"Burdett's Hospitals and Asylums of the World" is the only one and the price is £8 8s. "Vera" might see a copy at a public library possibly.

*Nursing Work in Ireland.*

(4) I wish for nursing work in the neighbourhood of Dublin, amongst the poor, if possible; surgical cases preferred. How can I get it?—*Nurse, Ireland.*

Why not apply to Miss Dunn, the Superintendent of the Irish Branch of the Queen Victoria's Jubilee Nurses Institute, or advertise in our columns?

*The Children's Nurse.*

(5) Will Miss Lilian A. send address? This is always our rule with queries.

*Idiot Girl.*

(6) Can you tell me where an idiot (girl) can be received who is ineligible for Earlswood? A small sum weekly could be paid.—*M. E. N.*

We have made inquiries, and find that the workhouse is the only refuge for an idiot without means. You might enquire of the Metropolitan Asylums Board, Norfolk Street, Strand.

*Lectures to Probationers.*

(7) Kindly tell me if there is a book which would be of any assistance in giving lectures to probationers.—*M. A. C.*

"The Matron's Course," by Miss Orme. The Scientific Press, price 1s.

## Figures and Tables

**Figure f1:**
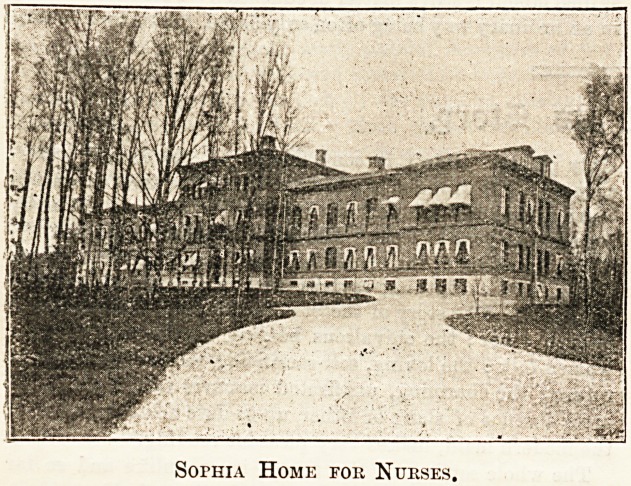


**Figure f2:**
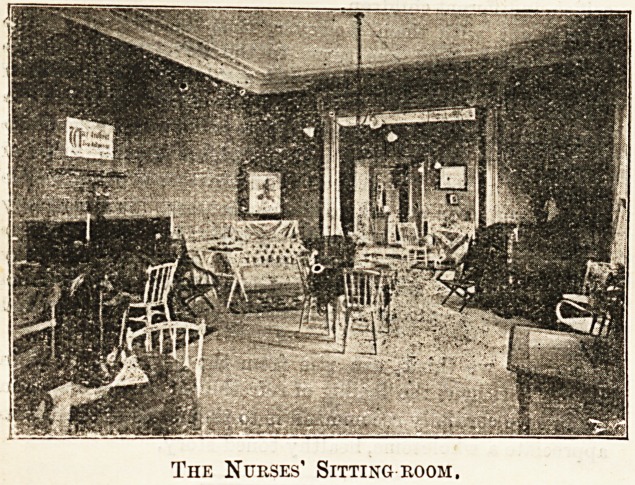


**Figure f3:**